# 1255. Single-tablet Regimens (STR) Offer Better Persistence and Adherence, with Lower Costs by Adherence Status, than Multiple-tablet Regimens (MTR) for People Living with HIV (PLWH) Enrolled in Medicaid

**DOI:** 10.1093/ofid/ofac492.1086

**Published:** 2022-12-15

**Authors:** Andrew P Brogan, Cindy Garris, Julie Priest, Victoria Divino, Jing He, Justin Chen, Mitch DeKoven

**Affiliations:** ViiV Healthcare, San Diego, California; ViiV Healthcare, San Diego, California; ViiV Healthcare, San Diego, California; IQVIA, Falls Church, Virginia; IQVIA, Falls Church, Virginia; IQVIA, Falls Church, Virginia; IQVIA, Falls Church, Virginia

## Abstract

**Background:**

Pill burden associated with antiretroviral multiple-tablet regimens (MTR) can impact adherence. The shift to single-tablet regimens (STR) has lagged for people living with HIV (PLWH) covered by Medicaid. This study examines persistence, adherence, healthcare resource utilization (HCRU), and costs by STR or MTR use for new initiators and treatment-experienced PLWH over a 1-year study period.

**Methods:**

A linked patient population was applied using data from IQVIA’s Prescription Claims (Rx), Professional Fee Claims (Dx), and Hospital Charge Data Master (CDM). A 6-month pre-index period was used to assess study eligibility and baseline characteristics. A 12-month post-index period was used to descriptively evaluate treatment patterns and HCRU/costs. Two mutually exclusive cohorts were created based on STR or MTR use during the selection window (01/2018-07/2019). For the STR cohort, date of the first STR claim during the selection window was termed the index date. For the MTR cohort, the date of the first MTR drug during the selection window was termed the index date.

**Figure:**
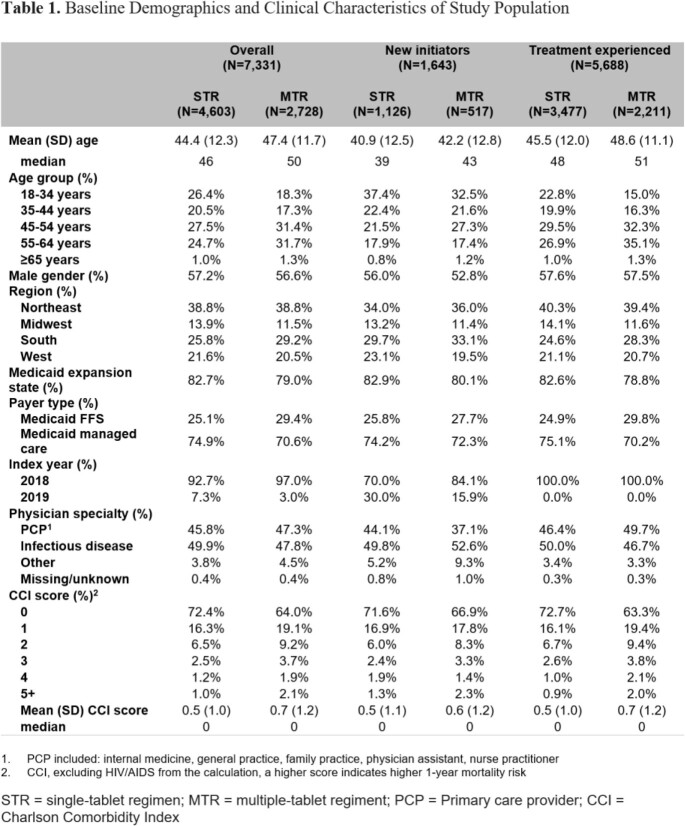

**Results:**

The final sample comprised 4,603 PLWH in the STR cohort and 2,728 in the MTR cohort (Table 1). The proportion persistent over the 1-year follow-up was higher among treatment experienced compared to new initiators, and higher for STR compared to MTR (Figure 1A). The proportion adherent was higher among treatment experienced compared to new initiators, and higher for STR compared to MTR (Figure 1B). HIV-specific per member per month (PMPM) pharmacy costs were higher among treatment experienced compared to new initiators, and higher for MTR compared to STR (Figure 2). Adherent PLWH had a lower proportion with ≥ 1 all-cause emergency room visit compared to non-adherent PLWH within a cohort/treatment status category; minimal differences in ≥ 1 all-cause hospitalization. Adherent PLWH had higher mean all-cause costs than non-adherent PLWH, driven by pharmacy costs. STR PLWH had lower mean all-cause total costs compared to MTR PLWH with the same adherence/treatment experience status (Figure 3).

**Figure:**
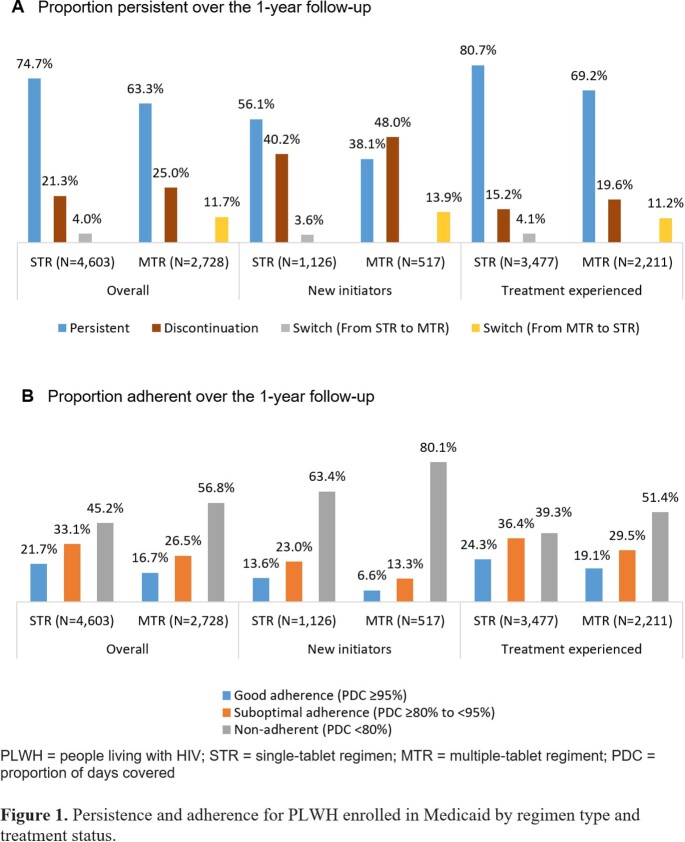

**Figure:**
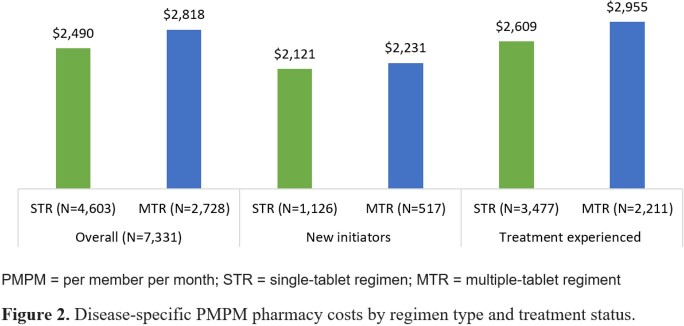

**Figure:**
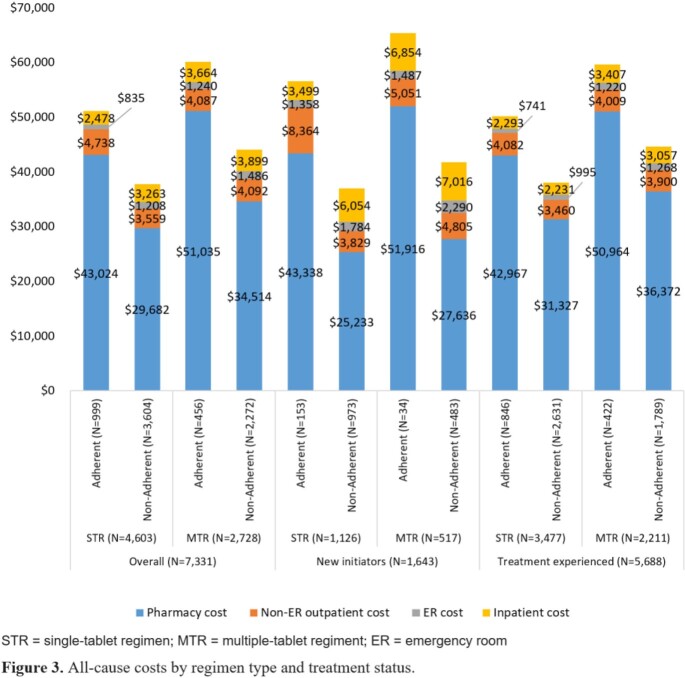

**Conclusion:**

PLWH enrolled in Medicaid are more persistent and adherent to STR than MTR. Among PLWH adherent to antiretroviral therapy, STR offer potential cost savings over MTR for appropriate patients.

**Disclosures:**

**Andrew P. Brogan, PhD**, ViiV Healthcare: Employee, Salary|ViiV Healthcare: Stocks/Bonds **Cindy Garris, MS**, ViiV Healthcare: Employee|ViiV Healthcare: Stocks/Bonds **Julie Priest, MSPH**, ViiV Healthcare: Employee, Salary|ViiV Healthcare: Stocks/Bonds **Victoria Divino, BA**, IQVIA: Employee, Salary|ViiV Healthcare: Grant/Research Support **Jing He, PhD**, IQVIA: Employee, Salary|ViiV Healthcare: Grant/Research Support **Justin Chen, MHS**, IQVIA: Employee, Salary|ViiV Healthcare: Grant/Research Support **Mitch DeKoven, MHSA**, IQVIA: Employee, Salary|ViiV Healthcare: Grant/Research Support.

